# Patient perspectives on the role of orthopedic nurse practitioners: a cross-sectional study

**DOI:** 10.1186/s12912-024-02014-8

**Published:** 2024-06-05

**Authors:** Merav Ben Natan, May Revach, Or Sade, Yaniv Yonay, Yaron Berkovich

**Affiliations:** 1 Pat Matthews Academic School of Nursing, Hillel Yaffe Medical Center, P.O.B. 169, Hadera, 38100 Israel; 2 Tel Aviv University, Tel Aviv, Israel; 3 The Orthopedics B Department, Hillel Yaffe Medical Center, Hadera, Israel; 4 Technion Faculty of Medicine, Haifa, Israel

**Keywords:** Nurse practitioners, Image of nursing, Orthopedics, Satisfaction with care

## Abstract

**Background:**

The inclusion of nurse practitioners (NPs) specializing in orthopedics shows potential for improving the quality of care for orthopedic patients. A critical aspect of assessing the feasibility and acceptance of introducing NPs into orthopedic settings involves understanding patients’ perspectives on this role. This study aims to explore the receptiveness of orthopedic patients to treatment by orthopedic Nurse Practitioners (NPs). Additionally, it investigates potential associations between patients’ willingness to engage with NPs, their familiarity with the NPs role, perceptions of nursing, and satisfaction with orthopedic nursing care.

**Methods:**

This cross-sectional study involved patients admitted to an orthopedic department in a central Israeli hospital between January and February 2023. Data was collected using a questionnaire consisting of five sections, validated by content experts. Statistical analyses, performed using SPSS, included descriptive statistics, independent samples t-tests, Pearson correlations, and linear regression.

**Results:**

Orthopedic patient participants demonstrated a moderate willingness to undergo treatment by orthopedic NPs, with over two-thirds expressing strong openness. Patients displayed a high willingness for NPs to engage in various clinical tasks, albeit showing lesser enthusiasm for medication management and preoperative evaluation. Positive attitudes towards nurses and familiarity with the NP’s role emerged as significant predictors of patient receptiveness to NPs’ treatment.

**Conclusion:**

Patient acceptance of orthopedic NPs varies across different aspects of care. While there is overall willingness to receive care from NPs, these nuanced preferences should be considered when implementing NPs in orthopedic settings. Awareness and positive perceptions play crucial roles in shaping patients’ willingness to receive care from these NPs.

**Trial registration:**

The research doesn’t report the results of a health care intervention.

**Supplementary Information:**

The online version contains supplementary material available at 10.1186/s12912-024-02014-8.

## Background

Orthopedic healthcare presents a myriad of challenges globally, stemming from a scarcity of healthcare providers and an increasing demand attributed to longer life expectancies. Nurse Practitioners (NPs) specializing in orthopedics offer a promising solution to enhance patient care quality [1, 2]. While NPs are widely embraced in primary care settings in North America, their utilization in orthopedics remains underexplored, despite demonstrated competence across various orthopedic settings [3].

NPs play pivotal roles in addressing specialist shortages, improving patient access, and fostering interdisciplinary collaboration within healthcare teams [4, 5]. Innovative models such as the 1:1 NP and surgeon approach have shown promise in enhancing patient care delivery and reducing wait times [5]. Moreover, NPs act as crucial communication liaisons, facilitating efficient coordination within healthcare teams and enhancing patient experiences [4, 6]. Evidence suggests that NPs contribute to reduced patient stays through improved health outcomes and streamlined interdisciplinary coordination [4, 5].

Patient satisfaction is consistently high with NPs in specialty settings, surpassing physician care standards [5, 6]. Boman et al. [7] provide compelling evidence of NPs’ effectiveness in diagnosing and treating minor orthopedic injuries, emphasizing their valuable and comprehensive role in positively impacting healthcare outcomes.

Despite their effectiveness in specialty settings and evidence of high patient satisfaction, there is a notable absence of NP in the orthopedic specialty area in Israel, primarily due to regulatory barriers [8]. It has been claimed that patient acceptance and willingness for autonomous and complementary models of health care are fundamental to informing policy change [9]. Accordingly, patient acceptance and support are considered as a key facilitator of NPs implementation [10, 11]. Therefore, it is critical to explore the patient perspective regarding the introduction of the orthopedic NP role.

Several factors may affect patient acceptance of the NP role. Notably, some studies reveal limited public understanding of the NP role or the scope of their practice [12, 13]. The concern is that the general public may not fully grasp the extended scope of the NP’s role compared to other nursing professionals, including their ability to independently manage cases. This lack of awareness could potentially impede the acceptance of the NP role as an effective and valuable component of healthcare delivery, as suggested by several recent studies [12–14].

In addition, acceptance of NPs may be affected by the way nursing is perceived by society [15]. For example, if nursing is perceived as a less prestigious or less skilled profession, it may lead to an undervaluation of the advanced skills and capabilities that NPs bring to patient care. Similarly, if nursing is perceived as subservient or lacking in decision-making authority, this perception could hinder the acceptance of NPs as autonomous healthcare providers.

Moreover, as there is evidence of a positive correlation between satisfaction with nursing care and trust in nurses [16, 17], we assume that patient satisfaction with nursing care may potentially affect patient willingness to be treated by an NP. Positive experiences with nursing care can contribute to increased acceptance of advanced nursing roles as patients recognize the competence, communication skills, and patient-centered focus that nurses bring to the healthcare landscape.

## Methods

### Aims

Primary aim: To investigate the willingness of orthopedic patients to undergo treatment administered by an NP. Secondary Aim: To explore associations between patients’ familiarity with the NP role, their perception of the nursing profession, satisfaction with orthopedic nursing care, and their willingness to receive treatment from an orthopedic NP. By investigating patient perceptions and attitudes towards NPs in orthopedic care, we aim to provide valuable insights into the potential role that NPs can play in addressing the current challenges in orthopedic healthcare delivery. Moreover, by identifying factors such as familiarity with the NP role, the image of nursing, and satisfaction with orthopedic nursing care, our study sheds light on the key determinants of patient acceptance of NPs in this specialty area. Through our research, we hope to not only contribute to the existing literature on NPs in orthopedic healthcare but also inform policymakers and healthcare stakeholders about the importance of recognizing and integrating NPs into the orthopedic care team. By highlighting the positive impact that NPs can have on patient outcomes, access to care, and interdisciplinary collaboration, we aim to advocate for the expanded role of NPs in orthopedic healthcare settings.

### Design

This was a cross sectional study.

### Participants

Convenience sampling was utilized in this study to facilitate the selection of participants from the orthopedic department of the hospital during January and February 2023. The recruitment process targeted patients who were admitted to the orthopedic ward for scheduled surgeries and postoperative care. This sampling method was chosen due to practical considerations and logistical constraints, allowing for the efficient recruitment of patients based on their ease of access and availability within the specified time frame. The aim was to ensure a varied sample in terms of gender, age, nationality, and type of surgery among the patients included in the study. While convenience sampling may limit the generalizability of the findings to the broader population, it was deemed suitable for the specific aims and scope of the research. Inclusion criteria required patients to be adults (aged > 18 years) proficient in either Hebrew or Arabic, with the ability to speak, read, and comprehend the chosen language, and possessing the cognitive capacity to actively participate in the study. Exclusion criteria encompassed patients who did not provide consent or refused to participate in the research. Individuals facing communication barriers that impeded their ability to offer informed responses were also excluded. Additionally, patients admitted for psychiatric reasons or those with severe mental health disorders were ineligible for participation.

Sample size was estimated using the G-Power 3.1.2 (http://www.psycho.uni-duesseldorf.de/abteilungen/aap/gpower3/download-and-register) online website. In the G*Power 3.1.2 software, a two-tailed test of the correlation coefficient in the bivariate normal model was used with the following input parameters (an alpha [α] error probability of 0.05, the estimated effect size used in the calculation was 0.25 and power of 0.80) which yielded a sample size of 120 patients. The sample size was determined to provide adequate statistical power for the primary outcome measure, which focused on examining the relationship between patients’ willingness to be treated by an orthopedic NP and various predictors, including familiarity with the NP role, attitudes towards nurses, and satisfaction with nursing care. Throughout the study, efforts were made to ensure that the sample size remained sufficient to detect meaningful associations between variables of interest. However, no adjustments were made during the study period in terms of sample size or follow-up procedures.

### Study instrument

The questionnaire was specifically designed for this study to capture the perspectives of orthopedic patients. It consisted of five sections tailored to align with the primary and secondary aims of the research (See supplementary file). In Sect. 1, participants were presented with 11 items to provide background information and indicate their familiarity with the NP role. Response options included demographic variables such as age, gender, marital status, number of children, nationality, and education level. Participants were asked to select the appropriate response or provide the requested information.

Section 2 explored the participants’ perceptions of nursing and attitudes towards nurses using the Nursing Image Scale developed by Porter and Porter [18]. This section assessed participants’ perceptions of the nursing profession and characteristics of nurses. It involved ten pairs of bipolar adjectives reflecting features of the nursing profession (e.g., boring vs. interesting, characterized by routine work vs. characterized by diverse and challenging work, and does not allow creative approach vs. allows creative approach), and 10 pairs characterizing nurses (e.g., has low professional status vs. has high professional status, has limited knowledge in her field vs. has extensive knowledge in her field, and lacks professional authority vs. has broad professional authority). Participants ranked these on a Likert scale from 1 to 6. A score closer to 1 reflected the participant’s agreement with the statement on the right, while a score closer to 6 reflected agreement with the statement on the left. The reliability of the Nursing Image Scale in this study was 0.91 (Cronbach’s alpha).

Section 3 investigated participants’ satisfaction with nursing care provided in the orthopedic department, consisting of 2 items (e.g., “How satisfied are you with the nursing care in the orthopedic department? 1. Not at all, 2. Slightly satisfied, 3. Satisfied, 4. Very satisfied”).

Section 4 investigated participants’ willingness to be treated by an orthopedic NP using 3 items (e.g., “How willing would you be to receive treatment from a nurse practitioner?”). Participants ranked these items on a Likert scale from 1 to 6, where 1 – Completely unwilling and 6 – Very willing.

Section 5 explored participants’ willingness for specific clinical operations to be conducted by an orthopedic NP, encompassing 9 items (e.g., “Managing medications, including prescribing, monitoring, and discontinuing treatment”). Participants rated their agreement level regarding a nurse performing these tasks for them on a Likert scale from 1 to 6, where 1 indicates “Strongly Disagree” and 6 indicates “Strongly Agree. Of note, Sects. 3–5 were developed for this study.

The outcomes assessed in the questionnaire were aligned with the primary and secondary aims of the study, which focus on investigating patients’ willingness to receive treatment from orthopedic NPs and exploring associations with familiarity with the NP role, perceptions of the nursing profession, and satisfaction with orthopedic nursing care.

#### Validation process

The questionnaire underwent pilot testing with 20 patients to assess its reliability. The reliability of the questionnaire, measured using Cronbach’s alpha, ranged from 0.71 to 0.91. Additionally, the questionnaire was translated into Arabic using the back translation method to ensure linguistic accuracy.

### Procedure and ethical considerations

This study received approval from the Institutional Review Board and Clinical Medical Research Ethics Committee of the hospital under protocol HYMC-22-0110. Prior to the distribution of the printed questionnaires to potential participants, informed consent was obtained from each individual. Participants were briefed on the research objectives, and assurances of anonymity were provided. Each participant willingly signed an informed consent form before participating in the study. The questionnaire was designed to take approximately 15 min to complete. After data collection, participant information was securely stored and anonymized to ensure confidentiality. Data access was restricted to authorized personnel involved in the academic research project, in compliance with institutional ethical guidelines and regulations.

### Statistical analyses

The data analysis was conducted using the SPSS for Windows (version 29.0, SPSS Inc., Chicago) statistical software package. Descriptive statistics, including means, standard deviations, and percentages, were employed to characterize the sample. To examine the relationship between patients’ willingness to be treated by an orthopedic NP (primary outcome) and study variables, a linear regression analysis was conducted. This analysis aimed to identify predictors of patient willingness to undergo treatment administered by an orthopedic NP.

For secondary outcomes, including relationships between study variables, independent samples t-tests and Pearson correlations were utilized. These statistical tests were chosen to explore associations between variables such as participants’ familiarity with the NP role, perceptions of the nursing profession, satisfaction with orthopedic nursing care, and their willingness to receive treatment from an orthopedic NP. Statistical significance was set at a *p*-value of < 0.05 for all analyses.

## Results

### Sample characteristics

Our study comprised *N* = 100 participants. The average age of the participants was 55.4 years (SD = 19.37, range 18–92). The gender distribution showed that 60% were men and 40% were women. In terms of ethnicity, 74% identified as Jews, while 26% identified as Arabs. Marital status varied, with 68% of participants being married. Among the married participants, 81% reported having children, with an average of M = 2.74 children (SD = 2.05, range 1–11). Additionally, 19% of participants were without children. Educational backgrounds were diverse, with 42% having completed secondary education, 36% holding an academic degree, and the remaining 22% having completed elementary education (Table 1).

**Table 1:** Sociodemographic data of the research population

Variables	Categories	%	M	SD
Age			55.4	19.37
Gender	Men	60		
	Women	40		
Nationality	Jews	74		
	Arabs	26		
Children	Yes	81		
	No	19		
Number of children			2.74	2.05
Education	Elementary	22		
	Secondary	42		
	Academic	36		

### Primary aim: patient willingness to be treated by an orthopedic NP

Patients demonstrated a fairly high willingness to be treated by an orthopedic NP (M = 4.96, SD = 0.83). (To clarify, in Table 2, a mean score of 1 indicates a low willingness, while a mean score closer to 6 indicates a high willingness to be treated by an orthopedic nurse practitioner.) Over 67% expressed a strong willingness for themselves, their spouses, and their children. Regarding specific clinical operations, patients exhibited high willingness for orthopedic NPs to undertake tasks such as patient and family education (85%), patient admission to the department (78%), and patient preparation for the operating room (70%). However, willingness was lower for operations such as medication management (M = 62%) and preoperative evaluation and treatment of related medical issues (61%) (Table 2).

**Table 2:** Means, standard deviations, and ranges of the research variables

Variable	M	SD	range
Willingness to be treated by an orthopedic nurse practitioner	4.96	0.83	1–6
Image of nursing	4.72	0.75	1–6
Attitudes towards nurses	4.90	0.89	1–6
Satisfaction with nursing care provided in the department	3.45	0.72	1–4

### Secondary aim: associations between variables

Positive but modest associations were identified between willingness to be treated by an orthopedic NP and attitudes towards nurses (*r* = 0.22, *p* < 0.05), the image of nursing (*r* = 0.33, *p* < 0.01), and satisfaction with nursing care in the orthopedic department (*r* = 0.26, *p* < 0.01). Patients familiar with the NP role expressed notably higher willingness (M = 5.46, SD = 0.64) compared to those unfamiliar with the role (M = 4.67, SD = 0.64) [t=-5.39 (df = 94.16), *p* < 0.01]. Additionally, patients with a relative working as a nurse showed a greater willingness (M = 5.12, SD = 0.76) compared to those without such relatives (M = 4.67, SD = 1.07) [t = 2.24 (df = 80.94), *p* < 0.05]. Furthermore, a positive but modest association was observed between patients’ age and their willingness (*r* = 0.21, *p* < 0.05), indicating that older patients expressed a slightly higher level of willingness. It is important to note that while statistically significant, these correlations reflect only a modest level of association, and interpretations should be cautious.

### Linear regression analysis of predictors of patient willingness

A linear regression incorporating variables associated with patient willingness revealed that familiarity with the NP role, a positive image of nursing, and having a relative working as a nurse emerged as independent predictors of willingness to be treated by an orthopedic NP. This regression model explained 34% of the variance in patients’ willingness (Table 3).

**Table 3:** Linear regression analysis of predictors of the patient willingness to be treated by an orthopedic nurse practitioner

Variable	Beta	SE	*P*-value
Image of nursing	0.50	0.099	0.038
Attitudes towards nurses	0.05	0.101	0.570
Satisfaction with nursing care provided in the department	0.04	0.156	0.793
Awareness of the role of a nurse practitioner	0.62	0.228	0.008
Having a relative who is working as a nurse	0.44	0.150	0.005
Age	0.01	0.004	0.840

## Discussion

This study explored the willingness of orthopedic patients to be treated by an orthopedic NP. In addition, the study explored whether familiarity with the NP role, the image of nursing, and satisfaction with orthopedic nursing care, are associated with this willingness.

The findings from this study suggest a generally positive disposition towards the acceptance of orthopedic NPs among orthopedic patients. Patients expressed a fairly high willingness to being treated by an orthopedic NP, extending this willingness to their family members as well. The high willingness for specific clinical operations, such as patient and family education, patient admission, and patient preparation for the operating room, indicates a recognition of the potential roles and contributions of orthopedic NPs in these areas.

However, it is noteworthy that a lower level of willingness was observed for certain operations, notably medication management and preoperative evaluation and treatment of related medical issues. This variation in willingness across different tasks suggests that there may be specific aspects of the NP role that contribute to concern among the patients.

These findings align with a previous Israeli study that revealed an overall favorable public disposition towards the introduction of advanced nursing roles. However, a note of concern emerged, particularly regarding tasks such as connecting and disconnecting patients from respirators and performing vaccinations not prescribed by a physician, indicating a perceived distinction between nursing and medical procedures [19]. Notably, a similar concern was evident in a study by Parker et al. [20], where patients identified specific activities as unacceptable for NPs in primary healthcare. This concern possibly underscores a broader perception among the public regarding the delineation of roles and responsibilities between nursing and medical practices.

Familiarity with the NP role emerged as a predictor of patient willingness to be treated by an orthopedic NP. Similarly, in an Australian study, participants who expressed unwillingness to be treated by an NP showcased a deficiency in comprehending the NP’s role within the broader healthcare team [14]. It has been suggested that lack of awareness of the NP role could potentially impede the acceptance of NPs as skilled and valuable contributors to healthcare [11, 12]. In addition, it was found that having a relative working as a nurse predicted a higher willingness to be treated by an orthopedic NP. Individuals with a family member in nursing may have a better understanding and appreciation for the roles and capabilities of nurses. Familiarity with a nurse in the family may foster trust in the nursing profession, potentially influencing the patient’s acceptance of the NP role, even in specialized areas such as orthopedics [21].

Moreover, we found that the image of nursing predicted the patient’s willingness to be treated by an orthopedic NP, in consistence with a previous study [15]. A positive image of nursing could contribute to patients’ confidence in skills and abilities of NPs, influencing their willingness to seek and accept treatment from these healthcare professionals.

This study also found that higher patient satisfaction with nursing care in the orthopedic department was associated with higher willingness to be treated by an orthopedic NP. This finding supports our assumption that positive experiences with nursing care can contribute to increased acceptance of advanced nursing roles as patients recognize the competence, communication skills, and patient-centered focus that nurses bring to the healthcare landscape.

Finally, in our study, older patients expressed a higher willingness to be treated by an orthopedic NP. This finding contrasts with the findings of a recent Australian study conducted by Dwyer et al. [12], which reported that individuals aged 65 and above, in comparison to other age groups, demonstrated a reduced willingness to be attended to by an NP. Interestingly, the authors of the Australian study proposed that individuals over the age of 65 might harbor a stereotypical perception of the nurse’s subservient role to the physician. This discrepancy in preferences across age groups highlights the nuanced and evolving nature of patient perceptions, emphasizing the need for further exploration into the factors influencing patients’ acceptance of NPs, especially in specialized fields such as orthopedics.

It is crucial to recognize that without a clear understanding of the scope of care provided by orthopedic NPs, assessing patient acceptance of this role becomes challenging. Orthopedic patients are surrounded by a multitude of healthcare professionals, including orthopedic nurses, orthopedic surgeons, physical therapists, and others, each bringing their unique expertise to the Table [3]. This collaborative effort may inadvertently lead to confusion if the roles of each team member are not clearly defined [22]. Therefore, patient acceptance of orthopedic NPs may depend on the clarity surrounding the NP’s role within the Multidisciplinary Team (MDT). Efforts aimed at elucidating the scope of practice of orthopedic nursing and clearly defining the role of NPs within the MDT are essential steps toward enhancing patient acceptance of orthopedic NPs.

### Limitations

This study is subject to several limitations that warrant consideration. Firstly, as the study was conducted at a specific hospital, the generalizability of findings to the broader population of orthopedic in-patients is limited. Moreover, the use of a questionnaire introduces the potential for response bias, where participants may be inclined to provide responses that align with perceived expectations rather than expressing their genuine views. Additionally, the study’s participant composition predominantly represents the Jewish sector, thereby inadequately capturing the perspectives of the Arab sector. Furthermore, a crucial limitation lies in the study’s emphasis on perceptions rather than experience. While the study provides insights regarding patient perspectives, the absence of direct experiences in patient care may limit the depth of understanding.

## Conclusions

This study aimed to investigate the acceptance of orthopedic NPs among orthopedic patients, focusing on associations between patients’ familiarity with the NP role, perceptions of nursing, satisfaction with orthopedic nursing care, and willingness to be treated by an orthopedic NP. Our findings indicate a generally positive inclination among orthopedic patients towards the acceptance of orthopedic NPs, tempered by nuanced skepticism, particularly concerning certain aspects of the NP role. Notably, familiarity with the NP role, having a relative in nursing, a positive image of nursing, and satisfaction with orthopedic nursing care emerged as key influencers positively associated with patient willingness to be treated by an orthopedic NP.

The implications of these findings suggest the need for further exploration of patient and staff experiences in contexts where NPs work. Rather than focusing solely on targeted educational initiatives, future research could delve into qualitative aspects of patients’ acceptance of NPs, exploring the underlying reasons behind their attitudes and perceptions. This could involve investigating patients’ experiences with NPs, their interactions with healthcare providers, and their expectations regarding the role of NPs in orthopedic care. Additionally, qualitative research could uncover any barriers or facilitators influencing patients’ willingness to be treated by NPs, offering richer contextual understanding beyond the quantitative associations identified in this study.

In conclusion, this study sheds light on the dynamic interplay of factors influencing patient acceptance of NPs in orthopedic settings. These insights are valuable for healthcare professionals, educators, and policymakers aiming to promote and integrate advanced nursing roles into specialized care.

### Electronic supplementary material

Below is the link to the electronic supplementary material.

Supplementary Material 1

## Data Availability

The datasets used and analysed during the current study are available from the corresponding author on reasonable request.
