# Supplementary material for: Patient perspectives on the role of orthopedic nurse practitioners: a cross-sectional study
Source: BMC Nurs. 2024 Jun 5;23:383. doi: 10.1186/s12912-024-02014-8 (PMC11155137; doi:10.1186/s12912-024-02014-8)
Supplement: Supplementary file 1 — Supplementary Material 1 [file 12912_2024_2014_MOESM1_ESM.docx]

The questionnaire

Section 1:

Age______________

Gender 1. Male 2. Female

Marital Status 1. Single 2. Married 3. Divorced 4. Widowed

Number of Children_____________

Education 1. Elementary 2. High school 3. Academic

Reason for Hospitalization:__________________

What is your nationality? 1. Jewish 2. Arab

Department Not Hospitalized 1. Orthopedic A 2. Orthopedic B

Have you been treated by a clinical specialist before? 1.No 2. Yes

Are you aware of the role of a clinical specialist? .1 No 2. Yes

Section 2: Nursing Image Scale developed by Porter and Porter

Section 3:

Satisfaction with Nursing Care in the Orthopedic Department

1. Not satisfied at all 2. Slightly satisfied 3. Satisfied 4. Very satisfied

Satisfaction with the Care in the Orthopedic Department

1. Not satisfied at all 2. Slightly satisfied 3. Satisfied 4. Very satisfied

Section 4:

Interest in Clinical Specialist Nurse Services

| **Service Description** | **Scale: 1 (Strongly Disagree) - 6 (Strongly Agree)** |
| --- | --- |
| Treatment by a clinical specialist nurse for yourself | 1 2 3 4 5 6 |
| Treatment by a clinical specialist nurse for your wife | 1 2 3 4 5 6 |
| Treatment by a clinical specialist nurse for your children | 1 2 3 4 5 6 |

Section 5:

Explored participants' willingness for specific clinical operations to be carried out by an orthopedic NP Activities Performed by Orthopedic Clinical Specialist Nurses Worldwide

| **Activity Description** | **Scale: 1 (Strongly Disagree) - 6 (Strongly Agree)** |
| --- | --- |
| Performing preoperative assessments and treating accompanying medical issues to optimize surgical outcomes | 1 2 3 4 56 |
| Receiving patients into the department | 1 2 3 4 56 |
| Preparing patients for surgery | 1 2 3 4 56 |
| Receiving patients from surgery, immediate assessment and postoperative care, identifying and treating complications | 1 2 3 4 56 |
| Referring for laboratory tests, imaging, and medical and paramedical consultations | 1 2 3 4 56 |
| Medication management – prescribing, monitoring, and terminating treatment | 1 2 3 4 56 |
| Pain management | 1 2 3 4 56 |
| Providing initial lifesaving treatment in emergencies | 1 2 3 4 56 |
| Educating the patient and their family | 1 2 3 4 56 |
